# Cecal microbiota and mammary gland microRNA signatures are related and modifiable by dietary flaxseed with implications for breast cancer risk

**DOI:** 10.1128/spectrum.02290-23

**Published:** 2023-12-07

**Authors:** Diana Wu, Lilian U. Thompson, Elena M. Comelli

**Affiliations:** 1 Department of Nutritional Sciences, University of Toronto, Faculty of Medicine, Toronto, Canada; 2 Joannah and Brian Lawson Centre for Child Nutrition, University of Toronto, Toronto, Canada; University of Nebraska-Lincoln, Lincoln, Nebraska, USA

**Keywords:** gut-breast axis, flaxseed, microbiota, microRNA, breast cancer, lignan

## Abstract

The gut microbial ecosystem underlies physiological relationships between the gut and distal organs. Mechanisms remain elusive but rely at least partially on the production of a diverse set of absorbable metabolites and host gene expression regulation. Here we show that in female mice, gut cecal microbiota profiles are related to microRNAs (miRNAs) expressed in the mammary gland. A subset of these miRNAs were found to regulate genes involved in breast cancer-related processes, such as cell proliferation and migration. To determine if these relationships could be exploited toward the reduction of breast cancer risk, we studied if they are modifiable by dietary flaxseed (FS), a source of lignan secoisolariciresinol diglucoside (SDG) and alpha-linolenic acid (ALA)-rich oil (FSO), both with antitumor effects. Importantly, SDG, but not ALA, needs microbial processing to release bioactive metabolites. We found that the microbiota and mammary gland miRNA are related, and FS modifies these relationships toward an antioncogenic phenotype. FSO- and SDG-related miRNAs were found to be involved in different pathways and neither FSO nor SDG alone could recapitulate the effects of whole FS, affecting unique pathways related to extracellular matrix processing. These findings highlight the existence of inter-organ microbiota-miRNA relationships, show that dietary interventions interact to affect them, and suggest a novel route for breast cancer prevention.

**IMPORTANCE:**

Breast cancer is a leading cause of cancer mortality worldwide. There is a growing interest in using dietary approaches, including flaxseed (FS) and its oil and lignan components, to mitigate breast cancer risk. Importantly, there is recognition that pubertal processes and lifestyle, including diet, are important for breast health throughout life. Mechanisms remain incompletely understood. Our research uncovers a link between mammary gland miRNA expression and the gut microbiota in young female mice. We found that this relationship is modifiable via a dietary intervention. Using data from The Cancer Genome Atlas, we also show that the expression of miRNAs involved in these relationships is altered in breast cancer in humans. These findings highlight a role for the gut microbiome as a modulator, and thus a target, of interventions aiming at reducing breast cancer risk. They also provide foundational knowledge to explore the effects of early life interventions and mechanisms programming breast health.

## INTRODUCTION

The gut microbiota is a bacteria-dominated community of trillions of microorganisms that utilize dietary components to produce bioavailable metabolites that affect host health. These metabolites at least partially underlie the relationship between the gut and distal organs (1). The role of microbial flaxseed (FS)-derived enterolignans in the context of the gut-breast axis exemplifies this concept. FS is the richest dietary source of the lignan secoisolariciresinol diglucoside (SDG) (2). The gut microbiota is necessary to convert SDG into the enterolignans enterodiol (ED) and enterolactone (EL) via the complementary action of members of the genera *Bacteroides*, *Clostridium*, *Eubacteriaceae*, *Peptostreptococcus*, *Eggerthella*, and *Enterobacter* (3). In mice, taxa such as the genus *Lactobacillus* and the family *Coriobacteriaceae* have been positively correlated with serum ED and EL levels (4). In humans, dietary interventions with lignans significantly increase urinary enterolignan excretion and are positively associated with microbial taxa such as the genus *Ruminococcus*, *Roseburia*, and members of the family *Lachnospiraceae* (5). High concentrations of circulating lignans have been associated with reduced mortality for breast cancer in postmenopausal women (6 – 9). ED and EL are phytoestrogens that are thought to have protective effects in postmenopausal breast cancer, altering proliferation and apoptotic biomarkers as well as c-erbB2 expression in a clinical trial (10). Urinary EL has been inversely associated with proteins involved in pro-proliferative and apoptotic pathways, such as the PI3k-Akt pathway (11). Interestingly, we found that microbiota metabolic activity, including that of non-lignan metabolizers, is important for lignan processing (4). This aligns with the suggestion that the gut microbiota is a viable target to increase the effectiveness of breast antitumor drugs (12). Importantly, beyond providing lignans, FS is an important source of fiber, protein, and oil (FSO). FS fiber and oil have been shown to affect microbiota composition in the gut (13, 14). FSO, in particular, is among the highest plant-based sources of alpha-linolenic acid (ALA), which delays mammary tumor onset, reduces tumor growth, and tumor proliferation (15 – 17). Thus, breast anticancer properties of FS may be the results of the combined action of its components in the whole food, or the single components may act independently. Interestingly, we found that FS, FSO, and SDG provided in equal amounts as in FS, generate specific microRNA (miRNA) responses in the mammary gland (18). MiRNAs are short, non-coding RNAs that are epigenetic regulators of transcription and by extension, translation. MiRNAs are thought to regulate up to 90% of all genes by mainly targeting the 3′ untranslated region of target mRNAs, leading to degradation or inhibition of translation (19, 20). Modulation of miRNA expression may be a mechanism underlying host response to metabolic signals, including those generated from microbial metabolites. We recently showed that developmental patterns of mammary gland miRNAs may provide clues to their dysregulated role in breast cancer (21). Here, we determined if the mammary gland miRNome and the gut microbiota are related, if this relationship is modified by dietary FS, and if FS-associated miRNAs are altered in human breast cancer. Then, we determined if FSO and/or SDG components are responsible for FS effects.

## RESULTS

**Fig 1:**
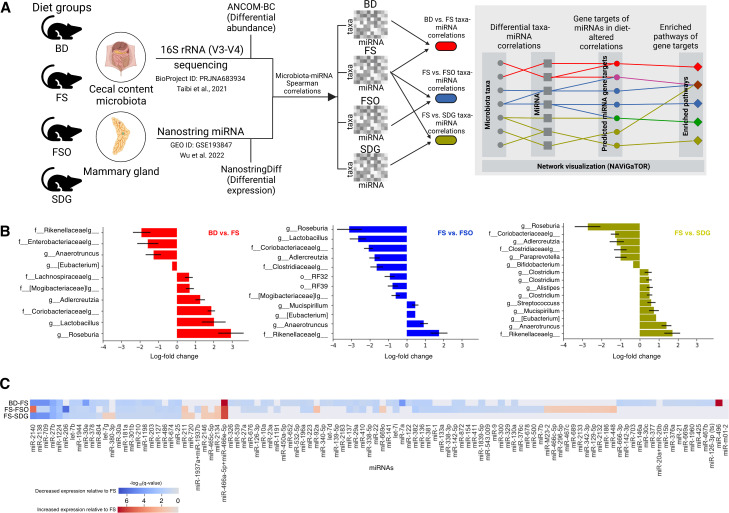
Approach to investigate the cecal microbiota-mammary gland miRNA relationship in mice fed a BD (control), FS diet, FSO diet, and SDG diet. (**A**) Fourteen C57BL/J mice were assigned to a BD, FS, FSO, or SDG diet. Cecal content and mammary glands were collected. Cecal content was Illumina-sequenced for the 16S rRNA V3-V4 region (23). Differential abundances of cecal content between diets were analyzed with ANCOM-BC. Mammary gland miRNA expression was quantified with Nanostring Technologies (24). Differential expression of mammary gland miRNAs was identified with NanoStringDiff (22). Relationships between cecal relative abundance and mammary gland miRNAs within diet were quantified with Spearman correlations with *n* = 5, 6, 5, and 5 for the BD, FS, FSO, and SDG diets due to paired-sample availability. Diet-induced differences were characterized by differential gene correlation analysis (DGCA) (25). Gene targets of miRNAs in diet-altered correlations were identified with miRdb (26), and enriched pathways using pathDIP (27). Panel (A) created with BioRender.com. (B) Differential abundance of taxa compared between BD vs FS (red), FS vs FSO (blue), and FS vs SDG (yellow) (left to right) diet groups. (**C**) Differential expression of mammary gland miRNAs relative to FS as identified with NanoStringDiff between the BD and FS diets, FS and FSO diets, and FS and SDG diets (top to bottom).

We used microbiota and miRNA data that we previously generated from the cecal content and mammary gland, respectively, of 3-week-old C57BL/6 mice receiving either a control basal diet (BD), or the same isocaloric diet modified to contain FS or its corresponding amount of oil (FSO), or SDG, for a total of four dietary groups (Fig. 1A). To investigate the effect of FS and its components on the gut microbiome-mammary gland miRNA axis, here, cecal microbiota data were analyzed by Analysis of Compositions of Microbiomes with Bias Correction (ANCOM-BC) and mammary gland miRNA expression data were analyzed via a generalized linear model to estimate fold-change using the dedicated package NanoStringDiff (22). Seventy-three taxa were identified at the genus level as represented in at least one of the four dietary groups (Table S1). Of these, 10, 12, and 15 taxa were found to be differentially abundant between the BD–FS, FS–FSO, and FS–SDG diet comparisons (Fig. 1B). Sixty-eight, 71, and 29 miRNAs were found to be differentially expressed between the BD–FS, FS–FSO, and FS–SDG diets (Fig. 1C).

### Cecal microbial taxa and mammary gland miRNA are linked

To understand if the cecal microbial taxa and mammary gland miRNA are linked, we identified significant Spearman correlations between the cecal microbiota and mammary gland miRNA expression in the BD group. One hundred and seventy one correlations were found to be significant (Fig. S1A). In the BD, 8 of the 10 most abundant taxa were found to be significantly correlated with miRNAs which targeted genes related to developmental processes such as collagen biosynthesis and modifying enzymes, developmental biology, transcriptional regulation of pluripotent stem cells, and protein digestion and absorption.

### FS, FSO, and SDG modify cecal microbiota-mammary gland miRNA correlations in a distinctive manner

In the FS, FSO, and SDG diets, respectively, 25, 139, and, 163 correlations between cecal microbiota and mammary gland miRNA were found to be significant (q < 0.05) (Fig. S1B through D). To determine if FS or its components affect the basal microbiota-miRNA relationship, we found correlations that were significantly changed between diet groups using differential gene correlation analysis (DGCA) (25). FS changed 5 BD correlations to positive, 6 correlations to negative, and 89 correlations were nullified (Fig. S2A). Of these changed correlations, the family *Enterobacteriaceae*, *Coriobacteriaceae*, *Lachnospiraceae*, and genus *Lactobacillus* were also found to be differentially abundant in response to FS (Fig. 1B, left). Compared to BD, FSO changed 4 correlations to positive, 79 to negative, and 80 were nullified (Fig. S2D) and SDG changed 4 correlations to positive, 63 to negative, and 73 were nullified (Fig. S2E). Family *Coriobacteriaceae* was found to be significantly decreased in the FSO and SDG diet compared to the BD and was also found in the differential correlations for both comparisons (Fig. S3).

### FS effects on gut microbiota-mammary gland miRNA correlations are not explained by its isolated FSO and SDG components

To investigate the extent to which FSO and SDG contribute to FS effects, we compared microbiota-miRNA correlations between the FS–FSO and FS–SDG diet groups. When compared to the FS group, only correlations between family *Ruminococcaceae* and miRNAs-140 and 30e were maintained in the FSO group; FSO changed 9 correlations to positive, 81 to negative, and 5 were nullified (Fig. S2B). Four taxa, including family *Clostridiaceae*, *Mogibacteriaceae*, order RF32, and genus *Adlercreutzia* (Fig. 1B, middle), and 14 miRNAs were also found to be differentially abundant between FSO and FS. When compared to the FS group, 1 microbiota-miRNA correlation was maintained in the SDG group; SDG changed 5 FS correlations to positive, 10 to negative, and 7 were nullified (Fig. S2C). Family *Clostridiaceae*, *Rikenellaceae*, *Coriobacteriaceae*, genus *Anaerotruncus*, and *Clostridium* in significantly changed correlations were also found to be differentially abundant (Fig. 1B, right) and 5 miRNAs were also found to be differentially expressed between SDG and FS. A comparison of FSO to SDG revealed 9 correlations changed to positive, 65 changed to negative, and 80 correlations nullified (Fig. S2F). No taxa were found to be significantly different between the FSO and SDG diet groups.

### FS alters relationships related to the PI3K-Akt-mTOR pathway

**Fig 2:**
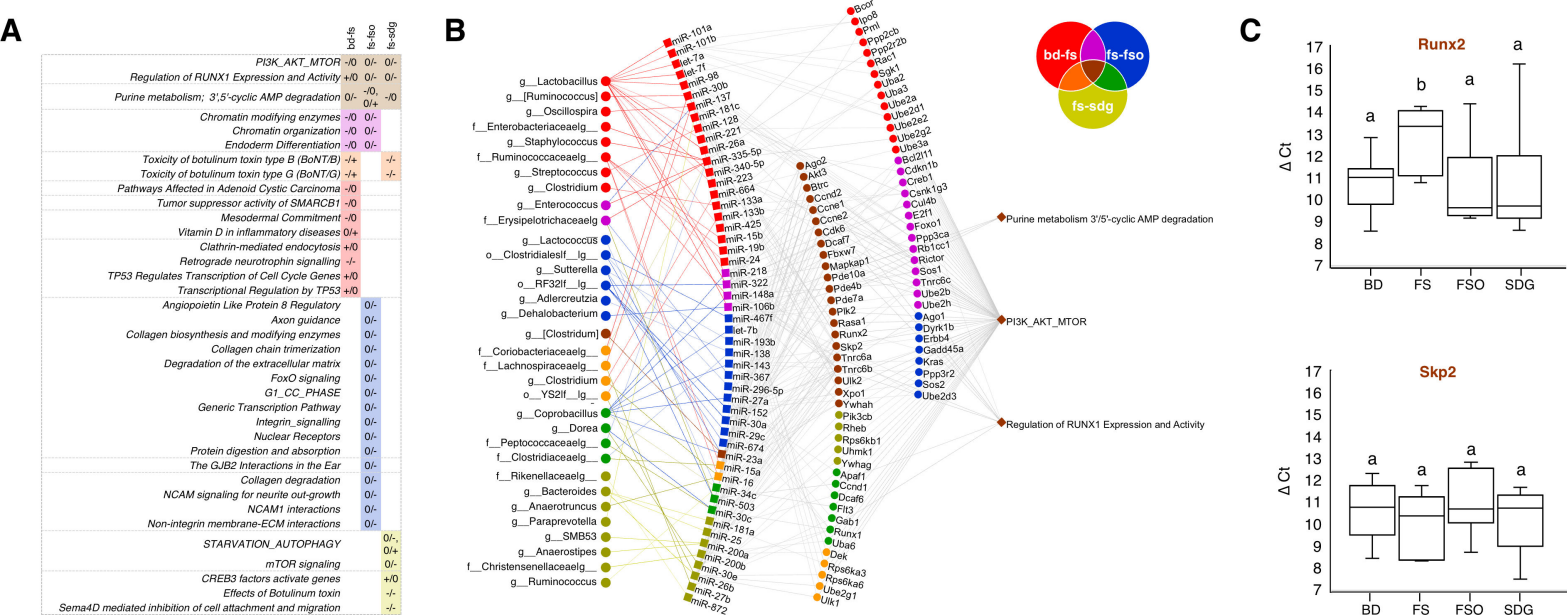
Shared and unique pathways significantly enriched by gene targets of miRNAs in diet-changed microbiota-miRNA correlations for comparisons between the BD–FS (red), FS–FSO (blue), and FS–SDG (yellow) diets. (**A**) Significantly enriched pathways (q < 0.05) in response to genes targeted by miRNAs (MiRTarget score >95) involved significantly changed correlations in the BD–FS, FS–FSO, and FS–SDG diet comparisons (q < 0.05). + indicates a positive correlation, 0 a null correlation, and − a negative correlation/indicates a correlation change between diets (e.g., −/0 indicates a change from − correlation in one diet to 0 correlation in another diet). (B) A network representation of the taxa-miRNA pairs with significantly changed correlations (q < 0.05) between diet groups and their gene targets identified with miRdb related to enriched pathways identified with miRDIP which were shared between the BD–FS, FS–FSO, and FS–SDG comparisons. Red: unique to the BD–FS comparison; blue: unique to the FS–FSO comparison; yellow/lime-green: unique to the FS–SDG comparison; purple: in common between BD–FS and FS–FSO comparisons; orange: in common between BD–FS and FS–SDG comparisons; green: in common between FS–FSO and FS–SDG comparisons; brown: in common between BD–FS, FS–FSO, and FS–SDG comparisons. (**C**) qPCR quantification of Runx2 (top) and Skp2 (bottom), members of the PI3k-Akt-mTOR pathway in the BD (*n* = 7), FS (*n* = 5), FSO (*n* = 8), and SDG (*n* = 8) diets. Groups that are significantly different (*P* < 0.05) are denoted with different letters (a–b) above each bar.

In order to understand the potential downstream effects of miRNAs involved in the diet-altered correlations, miRdb was used to predict miRNA gene targets. Predicted gene targets were used for gene enrichment pathway analysis with pathDIP, which integrates 23 pathway databases. Specifically, gene targets of the BD–FS, FS–FSO, and FS–SDG comparisons were investigated. Between BD and FS, 3750 gene targets of miRNAs in significantly changed correlations were found (Fig. S4A). Of these, 2012 were unique gene targets, 161 of which were involved in 16 significantly enriched pathways (Fig. S5, top). Between FS and FSO, 2903 gene targets of significantly changed miRNAs were found (Fig. S4B), with 1665 unique targets and 252 of these gene targets involved in 22 significantly enriched pathways (Fig. S5, middle). Between FS and SDG, 3996 gene targets of significantly changed miRNAs were found (Fig. S4C), with 2491 unique targets. Of the unique gene targets, 87 were involved in 11 significantly enriched pathways (Fig. S5, bottom). Three pathways were shared between the BD–FS, FS–FSO, and FS–SDG comparisons: the PI3K-Akt-mTOR, purine metabolism, and the regulation of RUNX1 expression and activity (Fig. 2A, top). A network was constructed to visualize these shared pathways (Fig. 2B). There were 22 genes in common between all three comparisons involved in the PI3K-Akt-mTOR and purine metabolism pathways (Fig. 2B) including Runx2 and Skp2. Specifically, Runx2 has been associated with spatial and temporal epithelial differentiation in the developing mammary gland and metastatic breast cancer (28). The taxa-miRNA correlation change was in the same direction relative to the FS group for the PI3K-Akt-mTOR pathway and the purine metabolism pathways (Fig. 2A). For the former, correlations changed from negative in the BD, FSO, and SDG diets to null in the FS diet. For the latter, correlations changed from null in the BD, FSO, and SDG diets to null in the FS diet.

### Gene expression related to the PI3K-Akt-mTOR pathway is reduced

To determine modulated miRNAs that may be affecting pathways altered by the FS diet, we found the miRNAs that both targeted genes involved in either the PI3K-Akt-mTOR or purine metabolism pathway pathways and were differentially expressed. In total, we found five, six, and four significantly differentially expressed miRNAs in the BD–FS, FS–FSO, and FS–SDG comparisons, which were involved in genes targeting either the PI3K-Akt-mTOR or purine metabolism pathways. Two of the miRNAs differentially expressed between BD and FS, miR-137 and miR-340-5p, were a part of the differential correlations related to the PI3K-Akt-mTOR pathways and were increased in the FS when compared to the BD diet (Fig. 1C). In the FS–FSO comparison, miR-137 was significantly lower in the FSO when compared to the FS diet (Fig. 1C). In the FS–SDG comparison, none of these two miRNAs were significantly differentially expressed (Fig. 1C). The miRNA expression was increased in FS with respect to all other diet groups (Fig. 1C). MiR-137 was predicted by miRdb to target Runx2 and miR-340-5p to target Skp2 (Fig. 2B). To determine expression levels of Runx2 and Skp2, we used quantitative PCR. We found significantly lower expression of Runx2 in the FS diet relative to the BD, FSO, and SDG diets (Fig. 2C, top). No other diet comparisons had significantly different expression of Runx2. Skp2 was not significantly differentially expressed between any diet groups (Fig. 2C, bottom).

### Flaxseed oil and SDG have unique roles in flaxseed effects

In order to determine shared or specific FSO and SDG effects as components of (in relation to) FS, pathways that were in common between the FS–FSO and FS–SDG comparisons, but not in the BD–FS comparisons were investigated. There were no pathways in common between the FS–FSO and FS–SDG comparison groups. To investigate how FSO may contribute to FS effects, pathways unique to the comparison between FS–FSO (not in the BD–FS or FS–SDG comparisons) were identified. There were 16 enriched pathways (Fig. 2A), 12 of which were also found in the BD–FSO comparison (Fig. S6). The pathways enriched included axon guidance, collagen synthesis and degradation, extracellular matrix signaling and degradation, and NCAM1 signaling. To investigate how SDG may uniquely contribute to FS effects, pathways unique to the comparison between FS and SDG were found. There were five pathways enriched (Fig. 2A). Pathways enriched included starvation autophagy, CREB3 factors, and mTOR signaling. There are 11 fewer pathways enriched compared to the FS–FSO pathways, potentially suggesting that there are fewer differences between the mechanism of action of FS and SDG when compared to FSO. Furthermore, the BD–SDG and FSO–SDG comparison also found the PI3k-Akt-mTOR pathway to be enriched, similar to the BD–FS comparison (Fig. S6).

### FS-associated miRNAs and predicted gene targets are altered in human breast cancer

**Fig 3:**
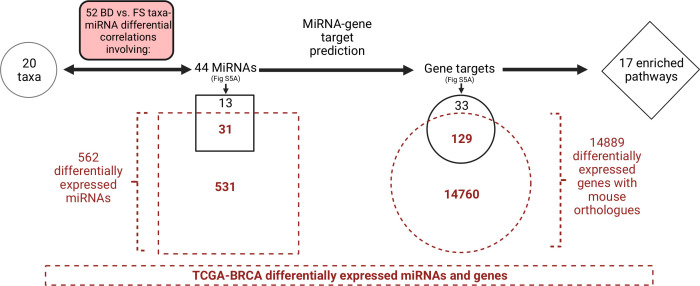
Number of significantly altered microbiota-miRNA correlations between the control BD and FS diets with miRNAs targeting genes within significantly enriched pathways in our mouse study. The TCGA-BRCA (https://portal.gdc.cancer.gov/projects/TCGA-BRCA) data set was used to determine differentially expressed miRNAs and genes between breast cancer tissues and matched normal samples. Mouse orthologues of differentially expressed miRNAs and genes were compared with those found in the differential correlation network between mice fed the BD vs FS diets. Created with BioRender.com

To investigate if the miRNAs and gene targets predicted may be relevant in human cancer, differential expression of publicly-available human miRNA- and RNA-Seq data between breast cancer and matched normal samples in The Cancer Genome Atlas (TCGA) (29) was determined with DESeq2; human–mouse orthologues were identified with the HGNC Comparison of Orthology Predictions Search (https://www.genenames.org/tools/hcop/). In breast cancer, 28,983/60,661 genes and 562/1,882 miRNAs were found to be differentially expressed (Table S2; Fig. 3). Of the 28,983 differentially expressed genes found in breast cancer, 14,889 had mouse orthologues (Fig. 3; Table S2). Out of the 161 genes involved in enriched pathways between BD and FS found in this study, 129 (80%) were found to be differentially expressed in breast cancer (Fig. 3), including significantly higher expression of Runx2 and Skp2 in both the BD and breast cancer groups (Table S2). Of the 44 miRNAs involved in enriched pathways between BD and FS, 31 were found to be differentially expressed in breast cancer (Fig. 3). Within these miRNAs, miR-137 and miR-340 expression were also decreased in breast cancer (Table S2).

## DISCUSSION

Compounds found in FS, such as SDG, require the microbiota to convert them into their bioactive forms which have been shown to have preventative benefits in breast cancer. These effects may act through mammary gland miRNAs, but the relationship between the gut microbiota and mammary gland miRNAs has never been explored. In this study, it was found that there is a relationship between the cecal microbiota and mammary gland miRNA and that this relationship is altered by an FS diet. The FS diet altered microbiota-miRNA relationships which targeted pathways related to the PI3K-Akt-mTOR pathway and genes previously found to be related to breast cancer. It was also found that within FS, FSO may act through different pathways than SDG, as a FSO diet had an increased number of altered correlations when compared to SDG, and FSO and SDG had no shared pathways related to altered correlations.

The PI3K-Akt-mTOR and Purine degradation/3′,5′ cyclic amp degradation pathways were enriched in response to FS. The PI3K-Akt-mTOR pathway has been implicated in increased ductal branching and TEB number in response to 0.1% arginine exposure in pubertal mice (30). TEBs are the most likely site for mammary tumors to develop, indicating a potential role of this pathway in pubertal development and cancer risk in later life. This pathway is activated in puberty and dysregulated in breast cancer (21). The PI3K-Akt-mTOR pathway has also been shown to play a role in breast cancer therapy resistance, with inhibition of mTOR resulting in restored sensitivity to tamoxifen (31). FS has previously been shown to have a synergistic effect on breast cancer with tamoxifen *in vivo*, with 10% FS and tamoxifen treatment significantly reducing tumor growth in comparison with tamoxifen alone (32). Previous and current clinical trials have tested inhibitors of mTOR (33) as well as upstream PI3K/Akt inhibitors (34).

Genes involved in the enrichment of the PI3K-Akt-mTOR pathway included Runx2 and Skp2, which are involved in mammary gland development and breast cancer. Runx2, a transcription factor, has been shown to promote phosphorylated Akt levels through mammalian targets of rapamycin complex-2 *in vitro* (35). Runx2 expression is reduced during late pregnancy, suggesting that it is necessary for full alveolar development in the mammary gland; moreover, loss of Runx2 expression increases mammary tumor survival (36). Furthermore, deletion of Runx2 has been shown to impair mammary stem regeneration, with a potential parallel to mammary tumors and their regenerative potential (37). Runx2 expression was found to be significantly decreased in the FS diet only, which suggests that miR-137 may be upregulated with an FS intervention and contribute to cancer-protective effects. Skp2 may also promote PI3K inhibitor resistance in aggressive breast cancer cells (38, 39). Runx2 and Skp2 are targeted by miR-137 and miR-340-5p, which were found to be significantly increased 2 and 1.8-log-fold, respectively, in the FS diet when compared to the BD diet (Fig. 1C). This corresponds to the change in correlation from negative in BD to nullified in FS. With a significant increase in miRNA, one might expect a change from a negative to a positive correlation between BD and FS. Here, a nullified correlation is observed, which may suggest that the miRNA reaches a steady-state maximal expression in response to microbial modulation from FS consumption. Further studies are needed to understand the time dynamics of this miRNA. The increased miRNA expression suggests that Runx2 and Skp2 may be downregulated in response to FS consumption. MiR-137 was associated with the family *Lachnospiraceae* and the genus *Oscillospira*, the former of which was found to be significantly enriched in the FS diet (0.66 log-fold change, q < 0.05). MiR-340-5p was associated with the genus *Lactobacillus* which was found to be enriched in the FS diet with a 2-log-fold increase (Fig. S1). *Lactobacillus* has been found to positively correlate with serum ED concentration (4). Species of the family *Lachnospiraceae* and genus *Lactobacillus* produce SCFA (40 – 42). However, Skp2 expression was found to be unchanged by diet, and thus may not contribute to these effects. Interestingly, we found that in human breast cancer samples, Runx2 and Skp2 are significantly increased and miR-137 and miR-340 are decreased, which is in line with the findings from the FS to BD diet comparison. In addition, 70% of miRNAs were found in differential correlations which led to altered pathways between BD and FS, and 80% of their gene targets were found to be differentially expressed in these breast cancer samples. When comparing differentially expressed miRNAs between BD and FS, only 37/59 ( ∼ 54%) were in common with those found to be differentially expressed in human breast cancer. This may indicate that FS drives benefits in the mammary gland during puberty which may have relevance to breast cancer, and these effects can be best elucidated when investigating miRNAs and genes within the context of their network connecting the microbiota and affected pathways.

Next, FS effects dependence on its FSO and/or SDG components was determined. To determine this, pathways enriched by correlation changes between the FS–FSO diets and FS–SDG diets which were not found in the BD–FS comparison were identified. There were no correlation changes in common between the FS–FSO and FS–SDG comparisons, suggesting that the FSO and SDG components have diverging roles in FS as a whole food. Indeed, FSO and SDG act through different mechanisms. SDG is digested by the gut microbiota to form ED and EL, bioactive molecules implicated in mammary gland development, breast cancer, and cardiovascular disease (43). Rather than digestion by the microbiota, FSO is rich in ALA and is thought to influence factors that interact with cellular receptors or influence the composition of the cell membrane (44).

Many of the enriched pathways related to FS–FSO changed correlations were related to extracellular matrix and collagen synthesis, regulation, and degradation. During pubertal development, the development of TEB (45) and the direction of branching morphogenesis are aligned with collagen-1 orientation (46). Mammary tumor cells also respond to collagen density and alignment, with collagen facilitating invasion and metastases of tumor cells (47 – 50). Tumor-associated collagen signature-3 is known to be associated with recurrence and poor survival (51). Many of the miRNAs in the correlation changes targeting the collagen/extracellular matrix (ECM) pathways changed from no correlation in FS to a negative correlation in FSO, suggesting FSO may alter collagen synthesis, regulation, or degradation when compared to FS. This highlights the parallel role collagen may play between pubertal mammary gland development and cancer, both of which are characterized by increased cellular invasion and proliferation.

These findings support previous evidence that FS may provide maximum benefit to the mammary gland when consumed as a whole food (4, 18). The direction of correlation change related to the PI3K-Akt-mTOR pathway and related genes were the same for BD, FSO, and SDG relative to the FS diet (nullified in FS, negative correlation in the other diets), suggesting that the pathway is an FS-specific taxa-miRNA change. A previous paper from our group found that ED and EL production positively correlated with FS, but not BD, FSO, and SDG diets (4), which aligns with our finding that FS uniquely alters taxa-miRNA relationships. Although the FS and SDG diets have the same amounts of SDG and fiber, the ED and EL production with the SDG diet was lower than with the FS diet, which may contribute to a diminished effect from an FS-equivalent SDG diet (18). This may be due to the higher proportion of fermentable soluble fiber in the FS diet than in the SDG diet. Indeed, previous studies have found that urinary lignan excretion is lower in rats consuming SDG compared to FS, even at an equivalent amount (52). These differences warrant further study into the mechanism of difference between FS and its components, not only in fiber but also in protein. In mice fed the FS diet, protein processing functions were enriched in the microbiome (4); FS protein may have antioxidant properties and cardiovascular benefits (21). FS fiber, which includes both soluble and insoluble fiber, can slow FS transit through the gastrointestinal tract, potentially increasing the absorption time of the lignan and oil components. The results further highlight the need for more research to disentangle these relationships. This knowledge may help in designing specific dietary strategies including the provision of fiber or FS-based synbiotics for mammary gland benefits.

Overall, this study showed the cecal gut microbiota is related to mammary gland miRNAs and consumption of FS may modulate mammary gland development pathways which may reduce breast cancer risk in later life. This study confirmed that FS components, FSO and SDG, target different physiological pathways and elucidate potential biological mechanisms for these differences. This study is a foundation for mechanistic studies of how FS acts through the gut microbiota to alter steady-state mammary miRNA expression. This may provide novel guidance for functional food interventions.

## MATERIALS AND METHODS

### Approach

We used mammary gland miRNA expression and gut microbiota data that we have previously generated in a mouse study where female C57BL/6 mice at 4–5 weeks of age were randomized to one of four isocaloric diets (*n* = 14/group): (i) basal modified AIN-93G, (ii) 10%FS, (iii) 3.67% FSO, and (iv) 0.15% SDG for 3 weeks until sacrifice (23, 24). The diets were formulated using the AIN-93G diet as a basis so that the amount of FSO and SDG in diets II and III, respectively, would equal their amount in diet I (23). At sacrifice, mammary gland, cecum contents were collected from *n* = 8–14/group, selected based on body weight and representing different cages. Cecal microbiota data were obtained via 16S rRNA Illumina sequencing using primers targeting the V3–V4 region (available at the NCBI SRA database, BioProject ID: PRJNA683934) (23). For this study, relative abundances of operational taxanomic units (OTUs) were copy-number corrected using the ribosomal RNA operons database (rrnDB) (53). For copy numbers not found at the desired taxonomic level, the copy number of the first available higher rank was used. The correction was adapted from Jian et al. (54) and is described in equation 1 where is one taxon within a taxonomic level, is the corrected relative abundance of taxon i, $r_{i_{0}}$ is the uncorrected relative abundance of taxon i, is the copy number of the taxon i as found in rrnDB.


(1) $$r_{i}=\frac{\frac{r_{i_{0}}}{c_{i}}}{\sum_{j_{0}}^{n}\frac{r_{j_{0}}}{c_{j}}}$$

The mammary gland miRNome (578 miRNAs) data were generated using the nCounter Mouse v1.5 miRNA Expression Assay Kit and the NanoString Technology (NanoString Technologies, Seattle, WA, USA) as previously described (18, 24) and are available with the Gene Expression Omnibus ID GSE193847 (GEO ID GSE193847) (18).

### Microbiota and miRNA differential analysis

For the microbiota data, ANCOM-BC version 1.6.4 was used to identify differentially abundant taxa between diet groups with default parameters, structural zero detection, a prevalence cutoff of 0.2, a conservative variance estimator, and global test (55).

For the miRNA data, NanoString count data were normalized using the estNormalizationFactors function, and differential expression was assessed using a generalized linear model likelihood ratio test via the glm.LRT function in NanoStringDiff version 1.24.0. Benjamini-Hochberg corrected q-values of less than 0.1 were considered significant. NanoStringDiff is a differential abundance method developed in R specifically for NanoString data, which differs from other differential abundance methods such as DESeq and edgeR as it takes into account positive control and housekeeping genes from the NanoString assay (22).

### Correlation analysis

Mammary gland miRNome data and cecal microbiota data matched by mice were used, resulting in *n* = 5–6/group. The associations between paired microbiota and miRNA were first assessed via Spearman correlation within each dietary group. Correlations were conducted in Python version 3.9.5 using the scipy.stats library at the genus level, using the lowest available rank above the genus when genus information was not available. Then, microbiota-miRNA correlations were compared between diets using DGCA via the DGCA v.1.0.2 package in R (Benjamini-Hochberg q < 0.05) (25). There were six diet comparisons: BD–FS (where “–” is “compared to”), FS–FSO, FS–SDG, BD–FSO, BD–SDG, and FSO–SDG. For DGCA analysis, microbiota data were filtered to ensure presence of all taxa in at least one mouse per diet group. The Fisher z-transformation was used to transform Spearman correlation values and compare z-scores of microbiota-miRNA correlations from different diets. There were nine possible outcomes from DGCA when comparing correlations in different diets: change from negative to positive correlation (−/+); change from positive to negative correlation (+/−); change from no correlation to positive correlation (0/+); change from no correlation to negative correlation (0/−); change from positive to no correlation (+/0); change from negative to no correlation (−/0); no change from no correlation to no correlation (0/0); no change from positive to positive correlation (+/+); and no change from negative to negative correlation (−/−).

### MiRNA gene target identification

Predicted gene targets of miRNAs involved in microbiota-miRNA correlations significantly modified by diet were identified using miRDB version 6.0 (26). Predicted gene targets with a minimum MirTarget score of 95 were included in downstream analyses.

### Pathway enrichment analysis and visualization of findings

Mouse genes were mapped to human orthologs and their corresponding pathways were identified with pathDIP 4.0 (27). Pathways with q-values (Bonferroni) less than 0.05 were considered significant. Taxa-miRNA-pathway networks were constructed and visualized with NAViGaTOR version 3.0 (56).

### Real-time quantitative PCR

Total RNA extracted as described previously (18) was reverse transcribed using the TaqManMicroRNA Reverse Transcription Kit for miR-137 and the High-Capacity cDNA Reverse Transcription Kit (Catalog no. 4368814, Applied Biosystems, ThermoFisher Scientific) for genes. The expression of B2m (Assay ID: Mm00437762_m1), Runx2 (Assay ID: Mm00501584_m1), and Skp2 (Assay ID: Mm00449925_m1) was quantified in triplicate using the QuantStudio5 Real-Time PCR System (Thermo Fisher Scientific). Expression data were normalized to the endogenous control B2m. Differential expression was assessed via two-tailed Welch’s unequal variances *t*-test (*P*-value < 0.05). Data were presented as mean relative expression relative to the reference diet.

### Human miRNA-Seq and RNA-Seq data

Publicly available miRNA-Seq and RNA-Seq data from non-metastatic breast cancer patients (*n* = 1111) in The Cancer Genome Atlas (TCGA-BRCA data set) (29) and matched normal samples (*n* = 113) were retrieved (https://tcga-data.nci.nih.gov/tcga/). Differential expression of miRNAs and RNA were assessed using DESeq2 (57). MiRNAs and genes with q-values less than 0.05 were considered significant. Microbiota data are available at the NCBI SRA database, BioProject ID PRJNA683934 (23). MicroRNA data are available at the NCBI Gene Expression Omnibus, ID GSE193847 (24).

## SUPPLEMENTAL MATERIAL

Fig. S1 spectrum.02290-23-s0001.tif Spearman correlations between the cecal microbiota and mammary gland miRNA for the BD, FS, FSO, and SDG diets.

Fig. S2 spectrum.02290-23-s0002.TIF Summary of differential correlations between different diets.

Fig. S3 spectrum.02290-23-s0003.TIF The network of DGCA-identified microbiota-miRNA differential correlations, miRdb predicted gene targets, and pathDIP-identified enriched pathways for comparisons between the BD–FS, FS–FSO, and FS–SDG diets.

Fig. S4 spectrum.02290-23-s0004.TIF Summary of miRdb predicted gene targets of miRNAs in significantly changed correlations with a MiRTarget score >95.

Fig. S5 spectrum.02290-23-s0005.tiff Differential expression of microbial taxa as identified with Analysis of Compositions of Microbiomes with Bias Correction (ANCOM-BC).

Fig. S6 spectrum.02290-23-s0006.TIF PathDIP-identified pathways enriched by gene targets of miRNAs in significantly changed correlations.

Table S1 spectrum.02290-23-s0007.xlsx Copy-number corrected microbial taxa data at the genus level.

Table S2 spectrum.02290-23-s0008.xlsx Differentially expressed miRNAs and genes as identified with miRNA-Seq and RNA-Seq data from the TCGA-BRCA data set between breast cancer and matched normal samples.

ASM does not own the copyrights to Supplemental Material that may be linked to, or accessed through, an article. The authors have granted ASM a non-exclusive, world-wide license to publish the Supplemental Material files. Please contact the corresponding author directly for reuse.
