# Supplementary figures and images for: Cecal microbiota and mammary gland microRNA signatures are related and modifiable by dietary flaxseed with implications for breast cancer risk
Source: Microbiol Spectr. 2023 Dec 7;12(1):e02290-23. doi: 10.1128/spectrum.02290-23 (PMC10783090; doi:10.1128/spectrum.02290-23)

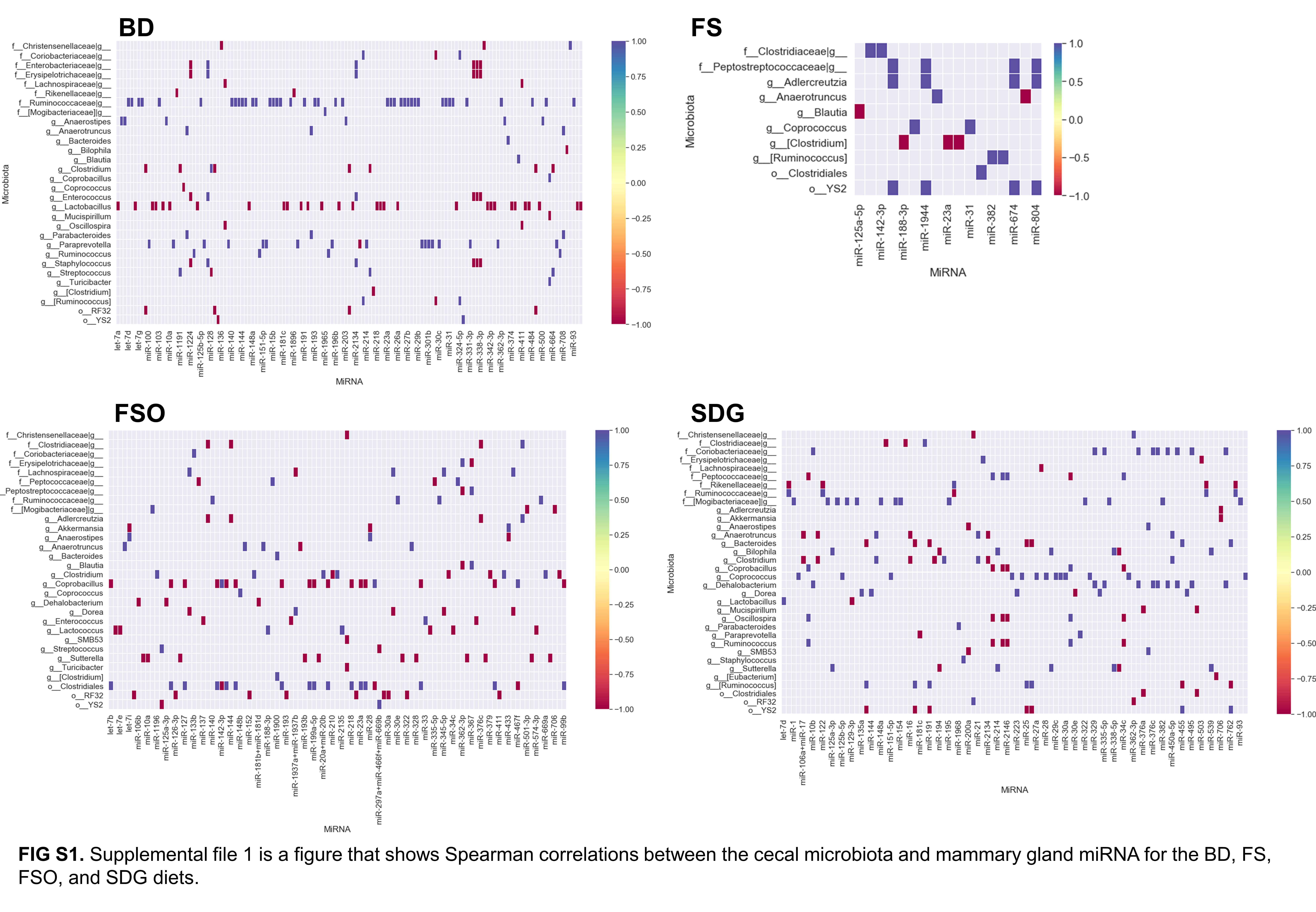

Supplement: Fig. S1 — Spearman correlations between the cecal microbiota and mammary gland miRNA for the BD, FS, FSO, and SDG diets. [file spectrum.02290-23-s0001.tif]

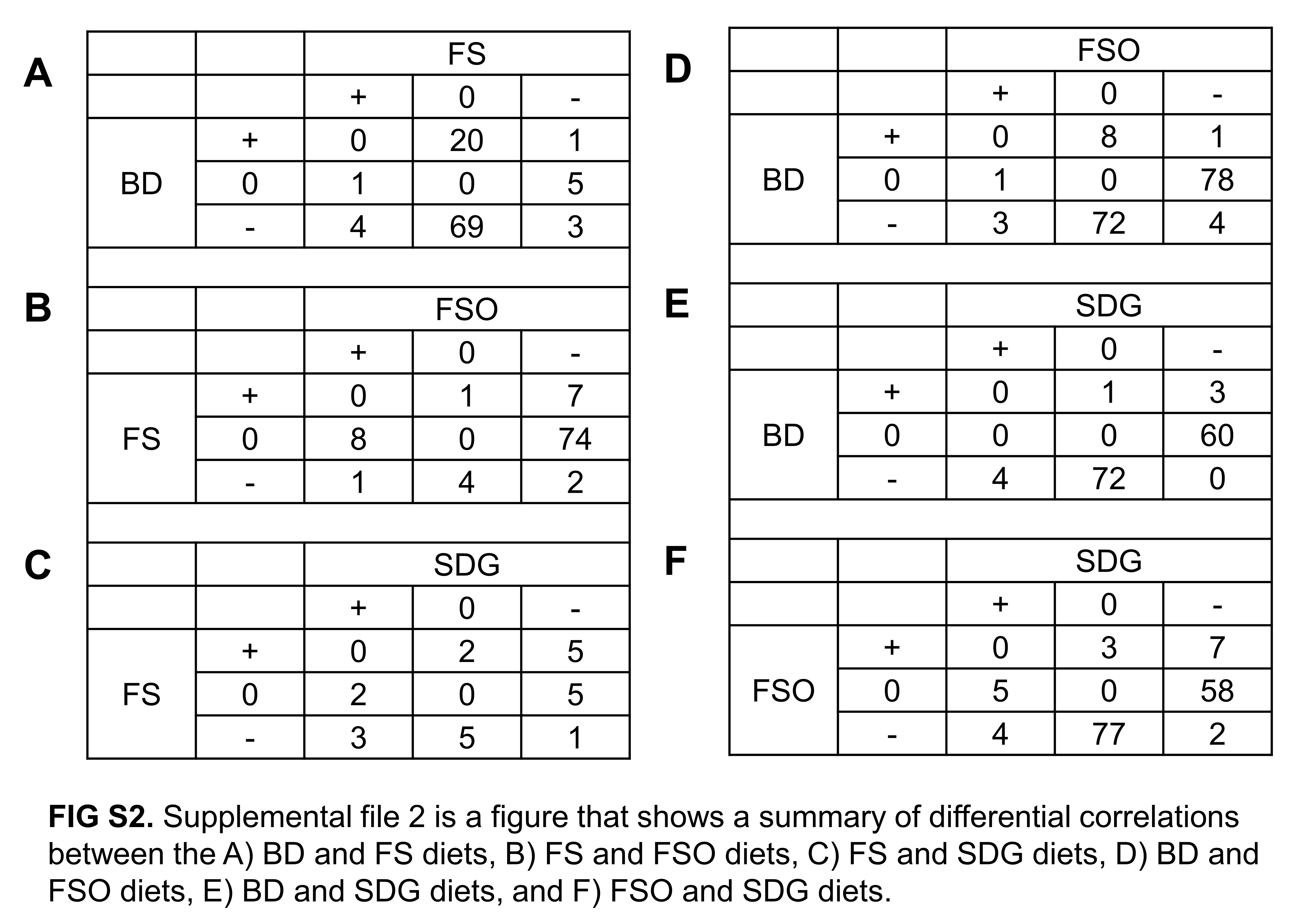

Supplement: Fig. S2 — Summary of differential correlations between different diets. [file spectrum.02290-23-s0002.tif]

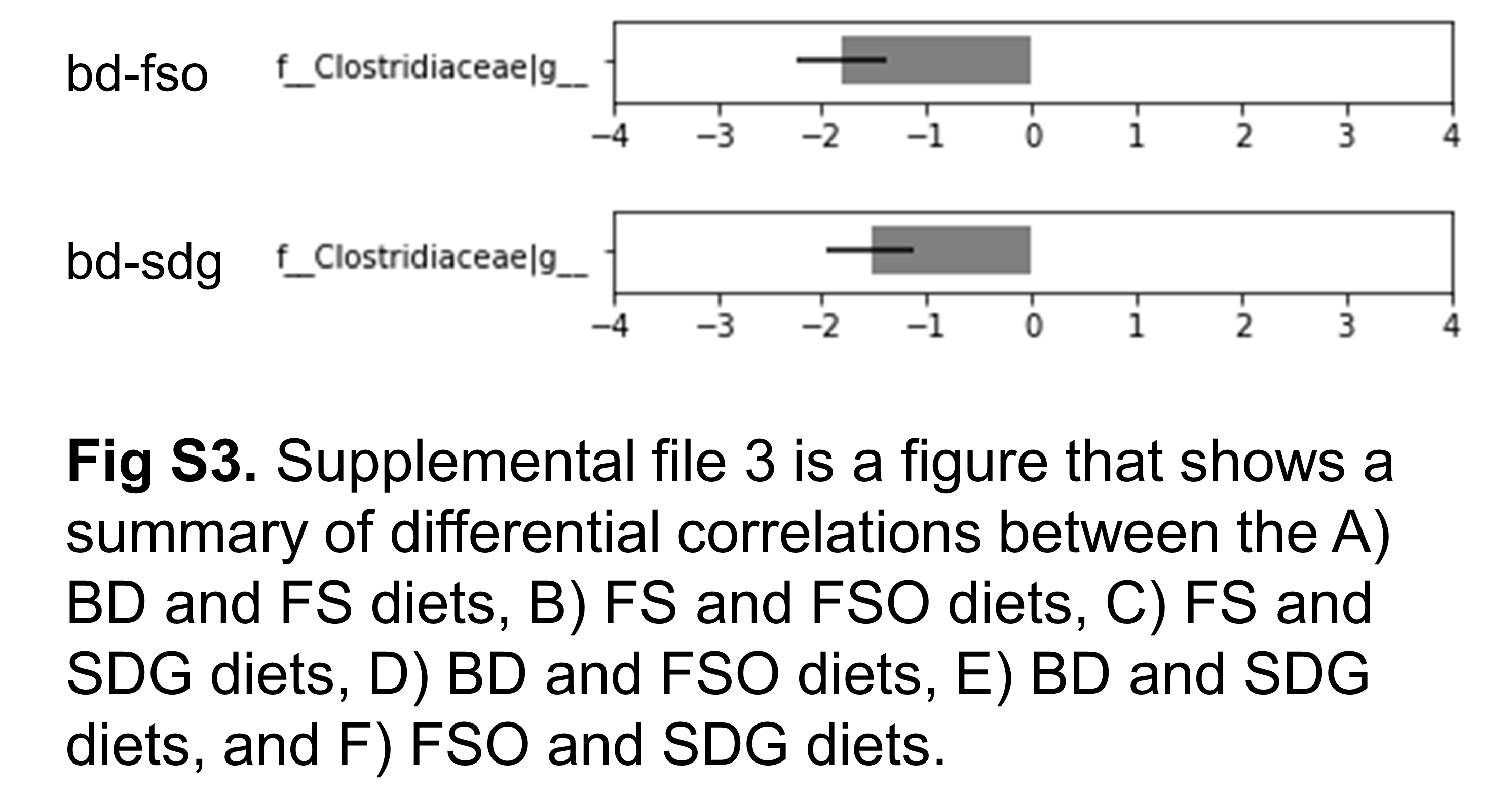

Supplement: Fig. S3 — The network of DGCA-identified microbiota-miRNA differential correlations, miRdb predicted gene targets, and pathDIP-identified enriched pathways for comparisons between the BD–FS, FS–FSO, and FS–SDG diets. [file spectrum.02290-23-s0003.tif]

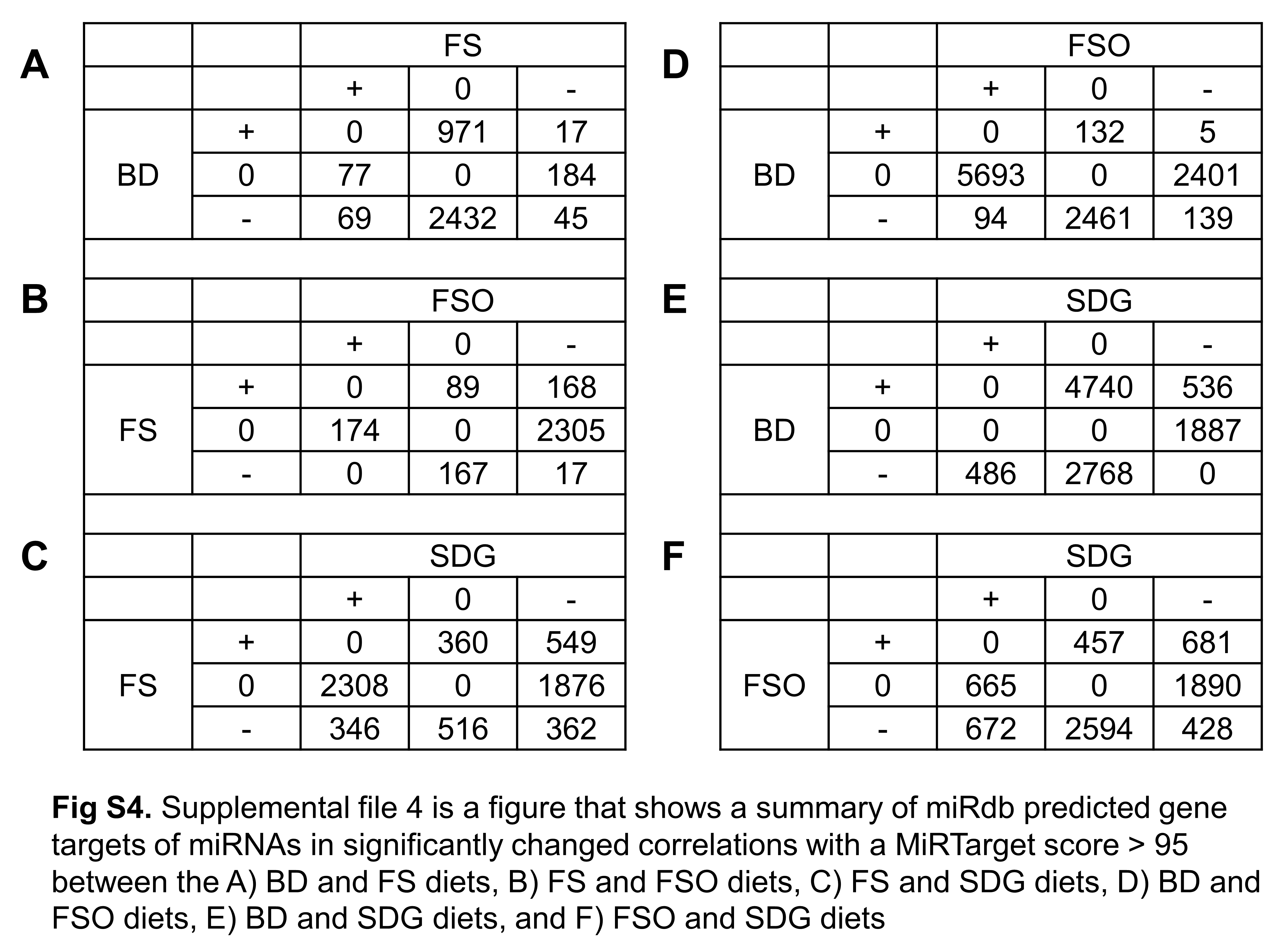

Supplement: Fig. S4 — Summary of miRdb predicted gene targets of miRNAs in significantly changed correlations with a MiRTarget score >95. [file spectrum.02290-23-s0004.tif]

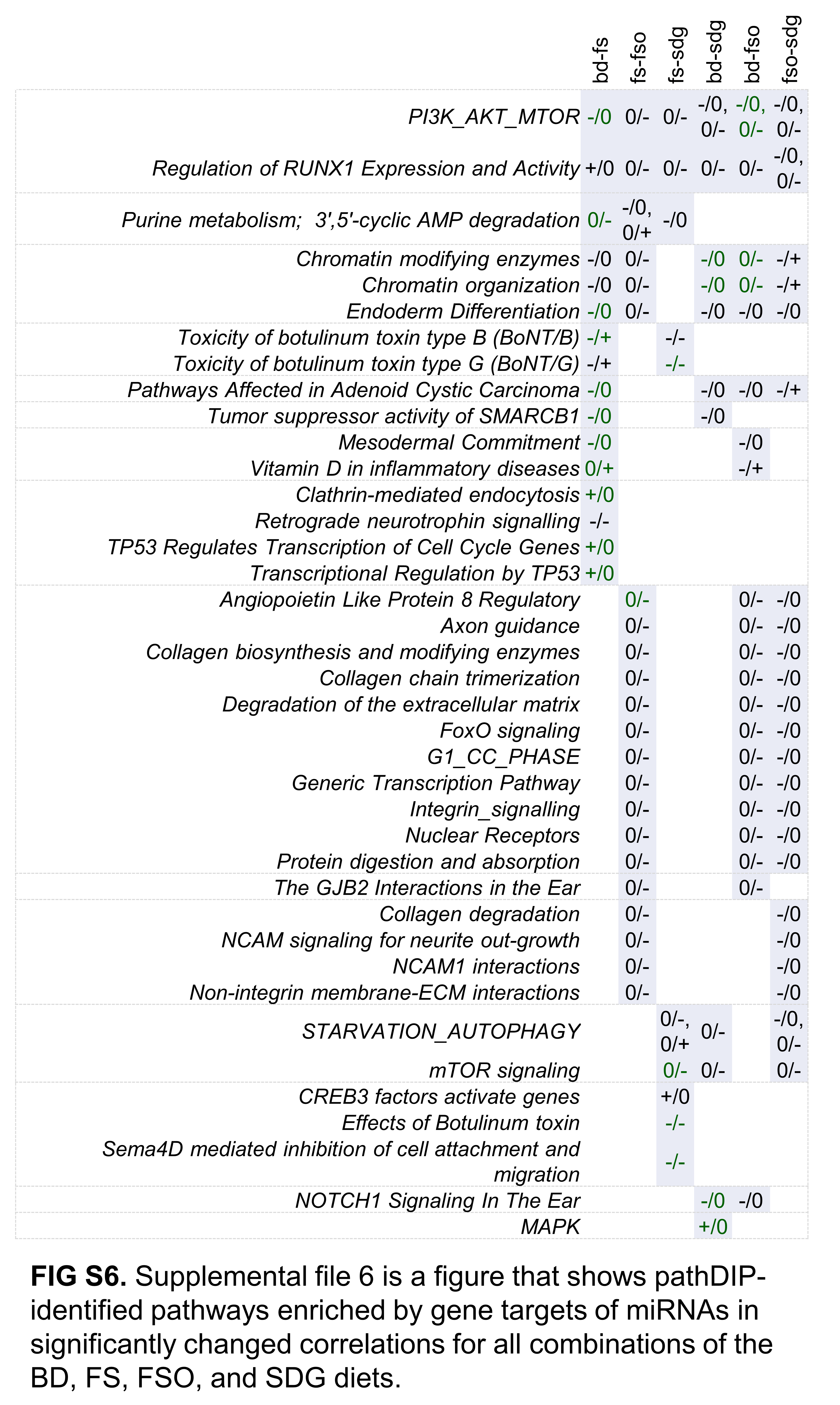

Supplement: Fig. S6 — PathDIP-identified pathways enriched by gene targets of miRNAs in significantly changed correlations. [file spectrum.02290-23-s0006.tif]
